# Modeling familial predictors of proband outcomes in neurogenetic disorders: initial application in XYY syndrome

**DOI:** 10.1186/s11689-021-09360-7

**Published:** 2021-03-22

**Authors:** Kathleen E. Wilson, Ari M. Fish, Catherine Mankiw, Anastasia Xenophontos, Allysa Warling, Ethan Whitman, Liv Clasen, Erin Torres, Jonathan Blumenthal, Armin Raznahan

**Affiliations:** Section on Developmental Neurogenomics, Human Genetics Branch, National Institute of Mental Health, National Institutes of Health, Building 10 Room 4N242 MSC 1367, 10 Center Drive, Bethesda, MD 20892-1367 USA

**Keywords:** Copy number variants, Sex chromosome aneuploidies, Neurogenetic disorders, Modeling penetrance, Precision psychiatry

## Abstract

**Background:**

Disorders of gene dosage can significantly increase risk for psychopathology, but outcomes vary greatly amongst carriers of any given chromosomal aneuploidy or sub-chromosomal copy number variation (CNV). One potential path to advance precision medicine for neurogenetic disorders is modeling penetrance in probands relative to observed phenotypes in their non-carrier relatives. Here, we seek to advance this general analytic framework by developing new methods in application to XYY syndrome—a sex chromosome aneuploidy that is known to increase risk for psychopathology.

**Methods:**

We analyzed a range of cognitive and behavioral domains in XYY probands and their non-carrier family members (*n* = 58 families), including general cognitive ability (FSIQ), as well as continuous measures of traits related to autism spectrum disorder (ASD) and attention deficit hyperactivity disorder (ADHD). Proband and relative scores were compared using covariance, regression and cluster analysis. Comparisons were made both within and across traits.

**Results:**

Proband scores were shifted away from family scores with effect sizes varying between 0.9 and 2.4 across traits. Only FSIQ and vocabulary scores showed a significant positive correlation between probands and their non-carrier relatives across families (*R*^2^ ~ 0.4). Variability in family FSIQ also cross-predicted variability in proband ASD trait severity. Cluster analysis across all trait-relative pairings revealed that variability in parental psychopathology was more weakly coupled to their XYY versus their euploid offspring.

**Conclusions:**

We present a suite of generalizable methods for modeling variable penetrance in aneuploidy and CNV carriers using family data. These methods update estimates of phenotypic penetrance for XYY and suggest that the predictive utility of family data is likely to vary for different traits and different gene dosage disorders.

**Trial registrations:**

ClinicalTrials.gov NCT00001246, “89-M-0006: Brain Imaging of Childhood Onset Psychiatric Disorders, Endocrine Disorders and Healthy Controls.” Date of registry: 01 October 1989.

**Supplementary Information:**

The online version contains supplementary material available at 10.1186/s11689-021-09360-7.

## Background

Disorders of gene dosage, ranging from aneuploidies to copy number variations (CNVs), are increasingly recognized as high-impact genetic risk factors for neuropsychiatric disease [1]. Recurrent pathogenic gene dosage disorders have been associated with increases in risk for several neuropsychiatric phenotypes, including autism spectrum disorder (ASD), bipolar disorder, and schizophrenia [2]. However, there is also strikingly high variability within carriers of any given aneuploidy [XYY [3]] or CNV [16p11.2 deletion [4], 22q11.2 deletion [5]], especially when carriers are identified through population-based versus clinical sampling frameworks [6, 7]. In particular, clinical sampling—as compared to population-based sampling—is influenced by referral and/or ascertainment biases that can result in an enrichment for greater phenotypic severity. The broad range of outcomes within aneuploidy and CNV disorders poses complex questions regarding the sources of phenotypic variation, and also frustrates personalized medicine approaches by making it difficult to predict outcomes in new cases. There has been growing interest in addressing this challenge by using family-based study designs to improve the prediction of penetrance for individual carriers, and to better understand sources of outcome variance across carriers.

Improved prediction of penetrance for individual carriers of rare genetic disorders is of great importance because information about the penetrance of a given genetic disorder (for example as used in a genetic counseling context) is typically based on reported phenotypic averages in clinical groups. However, given that there is typically significant phenotypic variability amongst individuals with any specific genetic disorder [4], a critical question for both families and clinicians relates to the likely phenotypic outcome for a given affected individual, which will reflect consequences of the genetic disorder as well as the genetic and environmental background upon which the disorder is occurring.

A recent study used family data to model outcomes in probands carrying a 16p11.2 deletion [4]. The authors reported significant moderate intraclass correlation between parent and proband scores for IQ, autism-related traits measured using the Social Responsiveness Scale (SRS [8]), and motor dexterity. This work demonstrates the potential for family-based study designs to improve outcome prediction in aneuploidy and CNV carriers. Important open questions remain, however, regarding: (i) whether the utility of family data in predicting proband outcomes might vary for different traits and different genetic disorders, (ii) whether there is cross-trait correspondence between family traits and proband outcomes, such as familial cognitive ability predicting proband psychopathology, (iii) whether one can boost prediction by adding data on early perinatal health to family trait measures, and (iv) whether trait correspondence between parents and offspring is itself modified by aneuploidy or CNV carriage.

Our current study develops and implements new analytic tools to address these open questions using data from XYY syndrome as proof of principle first application, although the approaches presented are explicitly designed to be generalizable to any aneuploidy or CNV disorder. XYY syndrome arises due to carriage of an extra Y-chromosome in males and is associated with increased height, decreased non-verbal and verbal IQ, and elevated risk for learning difficulties and social impairment [3, 9]. As a genetically defined condition that is associated with highly variable outcomes across individuals, XYY syndrome can be considered a paradigmatic example of a genomic dosage disorder that can impact human development. Our study design seeks to examine neurobehavioral trait correspondence between XYY probands and their non-carrier first degree relatives (parents and siblings) using four complementary analytic strategies.

First, we apply the standard approach of estimating Pearson correlation coefficients between XYY probands and their first-degree relatives for 12 different traits spanning cognitive and neuropsychiatric domains (see “Methods” section). This analysis gives useful trait-specific information, but also provides the broadest view to date regarding potential differences across neurobehavioral traits in observed covariation between carrier probands and their unaffected first-degree relatives. Second, we complement standard correlation analysis by also using a regression framework [10] to model proband-family interrelationships for each trait. Third, by extending this regression framework to a multivariate model, we test if knowledge of socioeconomic and perinatal variables can provide added prediction of variation in proband traits above and beyond knowledge of the trait value in unaffected family members. Given the well-established relationships between interindividual variation in IQ and psychopathology [11, 12], we also use multiple linear regression to test if family FSIQ is significantly associated with proband variation in non-FSIQ traits. Finally, we systematically map all cross-trait correlations across parent-proband, parent-sibling, and proband-sibling pairings to (i) directly capture differences in trait coherence between parent-proband and parent-sibling pairs, and (ii) find groupings of traits that show shared covariation in families. Inclusion of unaffected siblings provides a natural comparison for observed trait correspondence between parents and XYY probands in order to assess whether carriage of an extra Y-chromosome may not only alter phenotypes relative to family background, but also modify the concordance of trait variability across parents and their offspring.

The analytic framework presented is not only applicable to better understanding the sources of variation and predicting child-family outcomes in XYY syndrome, but it can also be used to determine the magnitude and variability of phenotypes associated with other aneuploidy and CNVs disorders. As discussed below, our findings carry implications for the use of family data to best predict outcomes in offspring carrying high-impact genetic risks for atypical development.

## Methods

### Participants

Our study population consisted of 58 families all defined through an index singleton XYY proband. Participants were recruited through the Association for X and Y Chromosome Variations [13] and the NIH Clinical Center Office of Patient Recruitment. The primary inclusion criterion was presence of a cytogenetically confirmed non-mosaic XYY karyotype in the proband and proband age between 5 and 25 years. Probands with very low birth weight were excluded given that this is not a recognized phenotypic association of XYY syndrome [14] and represents an alternative source of proband impairment that is not central to our analysis. We sought to include any biological parents and male siblings that accompanied the proband at enrollment. We restricted recruitment to male siblings to exclude sex as a source of outcome discordance between carrier probands and their siblings. Phenotypic data were available for unaffected full biological relatives as follows: proband mothers in 57 families, proband fathers in 34 families and proband siblings in 24 families (Table 1, Additional file 1). Informed consent (or assent where appropriate) was obtained from all study participants. All study procedures were approved by an NIH Institutional Review Board.

**Table 1:** Summary of clinical measures

	Probands (*N* = 58)	Siblings (*N* = 24)	Mothers (*N* = 57)	Fathers (*N* = 34)
*Mean* + *standard deviation (range)*				
Visit age (years)	12.6 + 5.39 (5.4–25.9)	13.4 + 6.1 (5.5–26.9)	44.8 + 7.0 (31.7–61.3)	44.0 + 5.5 (34.9–57.3)
FSIQ *N = 57/24/56/32*	86.65 + 13.96 (53–112)	104.17 + 14.24 (70–128)	106.3 + 9.27 (90–133)	109.0 + 11.76 (84–135)
Vocabulary *N = 45/23/56/32*	7.11 + 2.91 (1–14)	10.52 + 2.33 (5–15)	10.82 + 2.10 (8–17)	11.5 + 2.14 (8–16)
Matrix reasoning *N = 58/24/57/34*	8.97 + 3.05 (2–16)	11.29 + 3.44 (1–18)	11.53 + 1.99 (6–15)	11.82 + 2.69 (5–18)
SRS-2 Total *N = 58/24/57/34*	66.4 + 13.18 (42–100)	45.25 + 6.42 (37–66)	49.09 + 6.46 (39–66)	52.53 + 7.37 (40–76)
SRS-2 Awareness	66.67 + 13.21 (45–104)	48.62 + 10.37 (32–69)	52.37 + 5.91 (38–66)	55.91 + 7.1 (44–78)
SRS-2 Cognition	65.64 + 12.72 (44–100)	46.38 + 7.44 (39–70)	48.19 + 7.96 (37–65)	50.0 + 6.56 (39–70)
SRS-2 Communication	66.34 + 13.46 (40–96)	45.08 + 6.14 (38–64)	48.75 + 7.18 (38–75)	53.0 + 8.31 (41–78)
SRS-2 Motivation	60.33 + 12.05 (42–93)	46.46 + 7.56 (37–66)	51.68 + 9.12 (37–71)	54.62 + 10.01 (37–76)
SRS-2 RIRB	63.9 + 14.03 (41–100)	44.25 + 4.38 (40–58)	47.02 + 6.08 (40–70)	49.68 + 7.38 (40–72)
SRS-2 SCI	66.6 + 12.75 (43–99)	45.75 + 7.08 (36–68)	49.81 + 6.68 (39–68)	53.47 + 7.6 (40–77)
ADHD inattentive *N = 55/23/57/34*	73.15 + 12.61 (46–90)	55.78 + 11.42 (40–87)	48.26 + 15.32 (33–90)	48.06 + 14.35 (28–90)
ADHD hyperactive-impulsive	65.08 + 15.47 (40–90)	53.96 + 13.60 (35–86)	41.51 + 10.06 (29–79)	43.41 + 7.62 (32–62)

FSIQ is reported as a standard score. Vocabulary and matrix reasoning are reported as scaled scores. All other measures are reported as T-scores. *N* shows the number of participant values (proband/sibling/mother/father) for each measure. The SRS-2 subscales have the same *N* as the SRS-2 Total Score. The ADHD measures have the same *N* for both ADHD-traits. *FSIQ* Full Scale Intelligence Quotient*, SRS-2* Social Responsiveness Scale Second Edition. *SRS-2 Awareness* SRS-2 Social Awareness, *SRS-2 Cognition* SRS-2 Social Cognition*. SRS-2 Communication* SRS-2 Social Communication, *SRS-2 Motivation SRS-2 Social Motivation*, *SRS-2 RIRB* SRS-2 Restricted Interests and Repetitive Behaviors*, SRS-2 SCI* SRS-2 DSM-5 Social Communication and Interaction

### Testing and questionnaires

Group sizes ranged from 23 to 58 individuals per domain of measurement (see Additional file 2 for details). We included the following cognitive traits as measured using age-appropriate Wechsler scales: Full-Scale Intelligence Quotient (FSIQ-values scaled to have a population mean of 100 and a standard deviation (s.d.) of 15), vocabulary and matrix reasoning scores (scaled mean of 10 and a s.d. of 3). ASD-related traits were measured using the Social Responsiveness Scale Second Edition questionnaire (SRS-2 [15]). For this study, we analyzed the overall SRS-2 total score, scores for each of five treatment subscales (Social Awareness, Social Cognition, Social Communication, Social Motivation, and Restricted Interests and Repetitive Behavior), and one DSM-5 compatible subscale score (Social Communication and Interaction). SRS-2 T-scores have a scaled mean value of 50 (s.d. 10), with higher scores indicating greater impairment. ADHD-related traits were measured using two linked scales that together bridge the wide age-range of our study participants. Specifically, DSM scales for ADHD inattentive and ADHD hyperactive-impulsive symptoms were drawn from the Conners 3-Parent for participants under 18 years of age, and from the Conners’ Adult ADHD Rating Scales-Self-Report: Long Version (CAARS-S:L) for participants 18 years of age and older [16, 17]. Results are reported as *T* scores (scaled mean of 50 and s.d. of 10), with higher scores indicating greater impairment. We used a standardized clinical interview to gather data in probands on key perinatal variables that have previously been linked to neurodevelopmental outcomes: birth weight, gestation time, and maternal age [18–20]. We also included the sociodemographic questionnaire developed by the MacArthur Network, as socioeconomic status (SES) has also been linked to neurodevelopmental outcomes in the general population [21].

### Statistical analysis

**Fig. 1:**
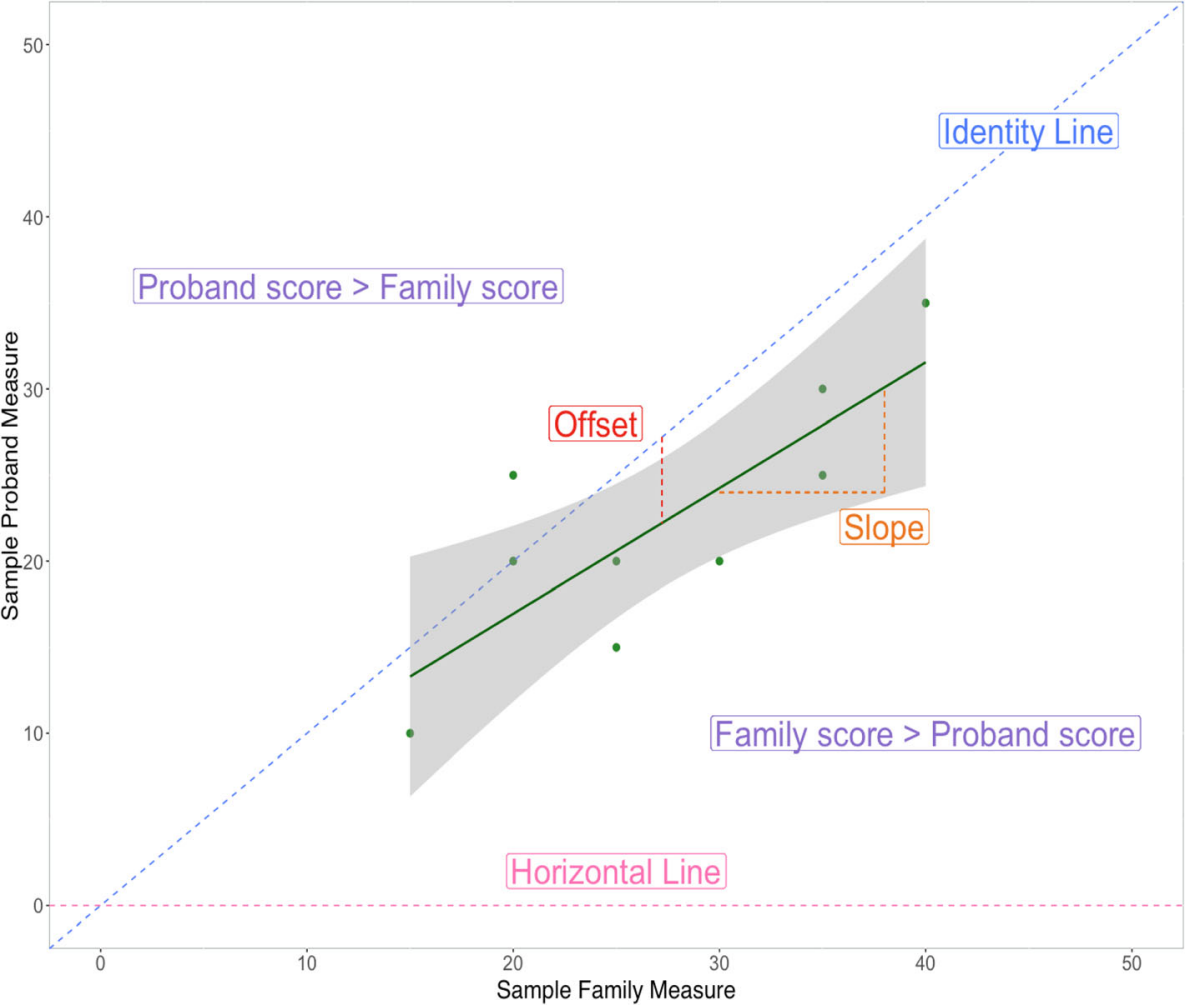
Annotated sample plot for simple linear regression. The univariate regression framework predicts proband outcome as a function of the family score for that measure. The model provides an offset (the difference between average family and average proband scores) and compares the regression slope to a horizontal line (*m* = 0) and an identity line (*m* = 1). *This plot is for visualization purposes only and does not include any data from the study*

To allow family-proband trait comparisons using all available families for all traits, we derived a summary “family score” for each trait by averaging scores from all available unaffected relatives (i.e., mother, father, sibling). For each of 12 traits, we first calculated proband-family trait correspondence across families using Pearson correlation coefficients. Next, we estimated trait-specific linear regression models to estimate an offset and slope for prediction of proband scores as a function of family scores (Fig. 1).

$$ Proband\_ Score= intercept+{\beta}_1\left( Family\_ Score\right)+e $$

For each trait, models were run after centering proband and family scores at the mean family score so that model intercepts would estimate the average score offset in probands relative to their unaffected family members (i.e., the penetrance of XYY for the trait in question). The regression framework above also quantified trait-specific regression slopes given by *ß*_*1*_ coefficient in the model above, which were compared against two reference values: 1 (the slope if proband scores scale linearly with family scores and show a stable offset across the range of observed family scores), and 0 (the absence of any significant linear relationship between proband and family scores). Because these regressions were calculated without prior standardization of proband and family scores, the observed slopes are not identical to the Pearson correlation between proband and family scores. Moreover, unlike correlation analyses, regression models (i) quantify the expected shift in proband scores for a unit shift in family scores in the unit of trait measurement, (ii) provide a means of testing if this regression coefficient differs from a pre-specified value of interest (Fig. 1), and (iii) provide a model for estimating the predicted proband score at a family score of interest (by definition, predicted proband offsets vary by family scores when the observed regression slope differs from 1). Given the small sample size of our study, and expectations from prior work that effect sizes for offsets would be significantly larger than those for the *ß*_*1*_ coefficient, we considered the *ß*_*1*_ coefficient tests to be exploratory in nature and in particular need for replication in larger samples. Furthermore, because we examine these slopes in a mass univariate fashion, our analysis do not explicitly test for differences in slopes for different traits.

We next extended these simple univariate regression models to determine if prediction of variation in a given trait across probands could be improved, beyond consideration of just that trait in family members, by incorporating additional knowledge of (i) family FSIQ (“Model 1” below) and (ii) a set of additional variables capturing socioeconomic and perinatal variables (“Model 2” below). Again, given the small size of our sample, these extended tests should be considered exploratory in nature.

***Model 1:***

$$ Proband\_ Score\_ TraitA= intercept+{\beta}_1\left( Family\_ Score\_ TraitA\right)+{\beta}_2\left( Family\_ FSIQ\right)+e $$

***Model 2:***

$$ Proband\_ Score\_ TraitA= intercept+{\beta}_1\left( Family\_ Score\_ TraitA\right)+{\beta}_2\left( Family\_ FSIQ\right)+\kern0.5em {\beta}_3\left( Family\_ SES\right)+{\beta}_4\left( Family\_ MacArthurLadder\right)+{\beta}_5\left( Proband\_ BirthWeight\right)+{\beta}_6\left( Proband\_ GestationLength\right)+{\beta}_7\left( Proband\_ MaternalAge\right)+e $$

The *ß*_*2*_ coefficient from Model 1 was compared to 0, to test for statistical evidence that variation in family FSIQ could explain significant variation in each proband trait beyond knowledge gained from the same trait in family members. Models 1 and 2 were compared using an ANOVA to first test for evidence that combined consideration of all specified socioeconomic and perinatal variables could predict additional variance in proband outcomes. A statistically significant increase in variance was required before consideration of individual *ß* coefficients from the socioeconomic and perinatal variables in Model 2. As the multiple linear regression models 1 and 2 could only be run with participants who had complete information on all predicting variables for each proband outcome, we also re-ran all univariate regression models with these reduced participant sets to allow direct comparison of the variance in proband outcomes that could be explained by univariate versus multiple linear regression models.

Finally, we extended our analysis to a simultaneous exploration of all possible pairwise trait interrelationships across and within the probands, siblings, and parents represented in our cohort. Specifically, we generated a square (36 × 36) correlation matrix of all pairwise Pearson correlations coefficients between 12 proband, sibling, and parent measures. For this analysis, a single parent score was calculated for each measure by averaging scores from the mother and father in each family, or using one parent’s data if only one parent participated. All measures were *z*-scored and FSIQ, vocabulary, and matrix reasoning were inverted, such that score polarity matched that for the other scales (i.e., lower scores indexing less impairment). The full correlation matrix was clustered using hierarchical agglomerative clustering, and selection of the final number of clusters (*k*) was determined using the elbow method [22].

*p* values for offsets, slope comparison to *m* = 0, slope comparison to *m* = 1, and *p* values of FSIQ contributing to proband outcome in the first multiple linear regression model are reported both with and without correction for multiple comparisons. *p* values surviving Bonferroni correction for multiple hypothesis testing across the 12 traits are starred (two-tailed). Analyses were conducted using RStudio 1.2.1335 [23] and the following packages: *purrr* [24], *dplyr* [25], *magrittr* [26], *ggplot2* [27], *viridis* [28], *heatmaply* [29], *cluster* [30], and *factoextra* [31].

## Results

### Participant characteristics

Descriptive statistics for trait scores in probands and non-carrier family-members are provided in Table 1, with complete demographic information presented in Additional file 1. Mean FSIQ in XYY probands was 86.65 + 13.96, and proband FSIQ ranged from 53 to 112. Total mean vocabulary and matrix reasoning scaled scores in probands were 7.11 + 2.91 and 8.97 + 3.05, respectively. In line with previous reports, mean scores for FSIQ, SRS-2, and Conners were all shifted relative to reference norms (decreased for FSIQ and increased for SRS-2, and Conners) in XYY probands, and we observed considerable variability in each trait across probands [32–34]. For example, proband SRS-2 total score ranged from 42 to 100 and proband ADHD hyperactive-impulsive symptoms ranged from 40 to 90. Trait scores in non-carrier family members were in the average range relative to population norms. For the 12 trait measures of interest, there were no significant differences between proband scores as a function of the availability of family data (i.e., the mean proband FSIQ score was not significantly different for the probands who had one relative participate versus those who had two or three relatives participate).

### Relationship between proband and family scores for individual traits: primary analysis of offset

**Fig. 2:**
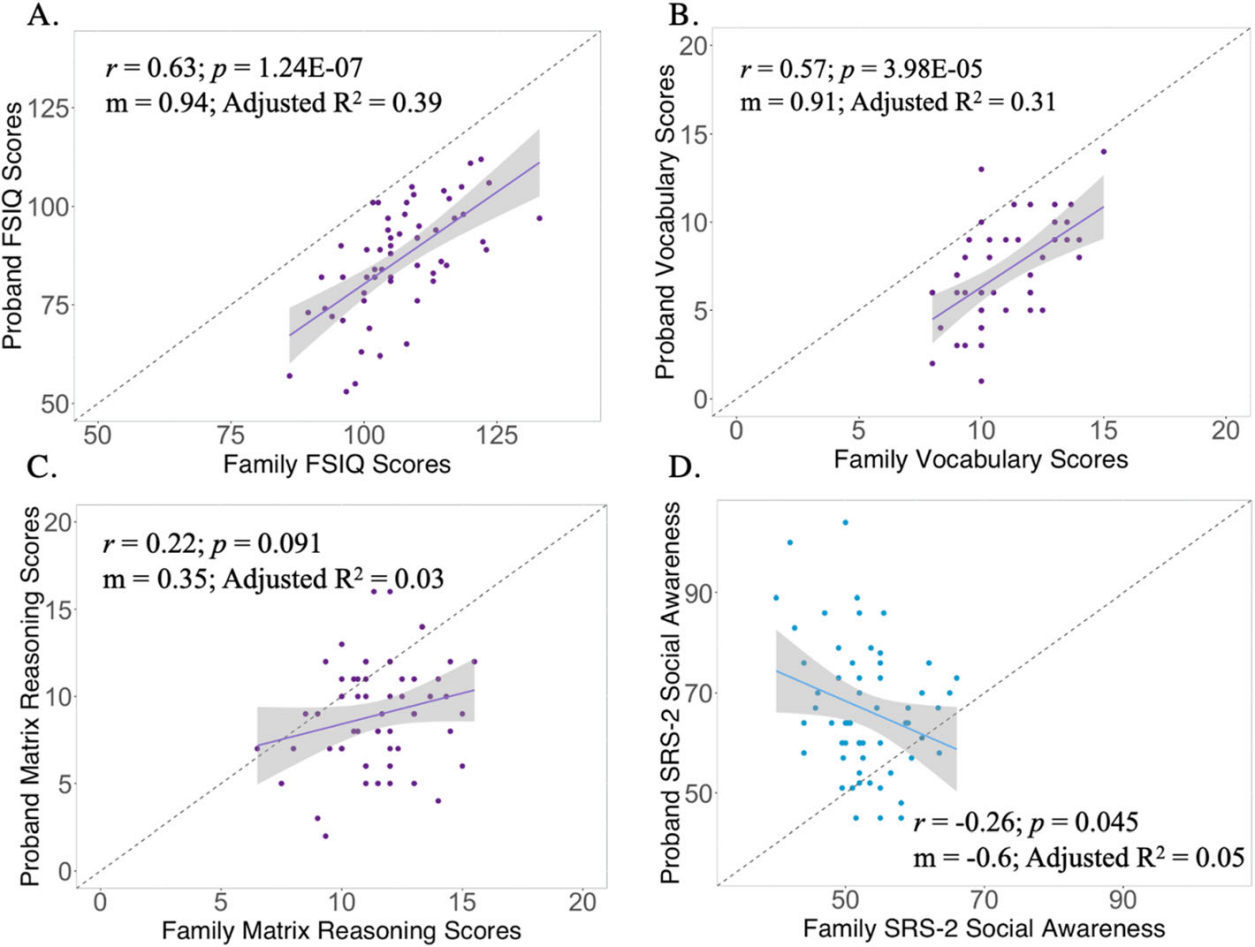
Proband to family univariate analysis. **a** Proband to Family FSIQ Scores. **b** Proband to Family Vocabulary Scaled Scores. **c** Proband to Family Matrix Reasoning Scaled Scores. **d** Proband to Family SRS-2 Social Awareness Scores. *Full Scale Intelligence Quotient (FSIQ). Social Responsiveness Scale Second Edition (SRS-2)*

Results from correlation and regression analysis of proband-family interrelationships for each trait examined are summarized in Table 2 and are graphically represented for selected traits in Fig. 2. The remainder of the trait-specific scatterplots are presented in Additional file 3. Observed correlations between probands and family scores varied greatly across traits. Statistically significant positive correlations between proband and family traits were only observed for FSIQ (*r* = 0.63, *p* < 0.0001) and vocabulary score (*r* = 0.57, *p* < 0.0001). Weaker (*r* < 0.3) and statistically non-significant positive proband-parent correlations were seen for matrix reasoning and ADHD hyperactive-impulsive symptoms. Proband-family correlation coefficients for SRS-2 total scores and subtests ranged from *r* = − 0.26 to *r* = 0.088; only the SRS-2 social awareness subscale reached statistical significance (*r* = − 0.26, *p* = 0.045).

**Table 2:** Summary of proband to family univariate analysis

Proband feature	Correlation coefficient (*r*)	Offset [*d*]; *p* value	Slope *m*, (s.d.)	*p* value of slope from *m* = 0	*p* value of slope from *m* = 1	Adjusted *R*^2^
FSIQ	*r =* 0.63	**−** 20.15 [**−** 1.34] ***p*** < 2E−16*	0.94 (0.15)	***p*** = 1.24E−07*	*p* = 0.684	*R*^2^ = 0.39
Vocabulary	*r =* 0.57	**−** 3.78 [**−** 1.26] ***p*** = 2.05E−13*	0.91 (0.2)	***p*** = 3.98E−05*	*p* = 0.662	*R*^2^ = 0.31
Matrix reasoning	*r =* 0.22	**−** 2.59 [**−** 0.86] ***p*** = 1.77E−08*	0.35 (0.2)	*p* = 0.091	***p*** = 0.003*	*R*^2^ = 0.03
SRS-2 Total	*r =* **−** 0.065	17.04 [1.7] ***p*** = 1.03E−13*	**−** 0.17 (0.34)	*p* = 0.629	***p*** = 0.001*	*R*^2^ = − 0.01
SRS-2 Awareness	*r* = **−** 0.26	13.83 [1.38] ***p*** = 3.67E−11*	**−** 0.6 (0.29)	***p*** **= 0.045**	***p*** = 1.17E−06*	*R*^2^ = 0.05
SRS-2 Cognition	*r* = 0.088	17.29 [1.73] ***p*** = 1.53E−14*	0.19 (0.28)	*p* = 0.51	***p*** **= 0.005**	*R*^2^ = − 0.01
SRS-2 Communication	*r* = **−** 0.039	17.185 [1.72] ***p*** = 1.68E−13*	**−** 0.09 (0.33)	*p* = 0.774	***p*** = 0.001*	*R*^2^ = − 0.02
SRS-2 Motivation	*r* = **−** 0.15	8.49 [0.85] ***p*** = 1.52E−06*	**−** 0.24 (0.21)	*p* = 0.26	***p*** = 2.60E−07*	*R*^2^ = 0.01
SRS-2 RIRB	*r* = 0.056	16.85 [1.69] ***p*** = 1.34E−12*	0.17 (0.41)	*p* = 0.678	***p*** **= 0.045**	*R*^2^ = − 0.01
SRS-2 DSM-5 SCI	*r* = **−** 0.085	16.45 [1.65] ***p*** = 1.02E−13*	**−** 0.2 (0.31)	*p* = 0.524	***p*** = 2.75E−04*	*R*^2^ = − 0.01
ADHD inattentive	*r* = 0.078	24.25 [2.43] ***p*** < 2E−16*	0.09 (0.15)	*p* = 0.57	***p*** = 2.17E−07*	*R*^2^ = − 0.01
ADHD hyperactive-impulsive	*r* = 0.22	21.85 [2.19] ***p*** = 9.42E−15*	0.45 (0.28)	*p* = 0.106	***p*** **= 0.049**	*R*^2^ = 0.03

The [*d*] is Cohen’s *d* effect size. The slope (m) is standardized residual indicating the effect size shift in proband score associated with a 1 standard deviation shift of family score. *m* = 0 (comparison to horizontal line). *m* = 1 (comparison to identity line). *p* value of slope from 0 also applies to null hypothesis test for Pearson correlation coefficient. (*) indicates survived multiple hypotheses testing Bonferroni correction. The relationship between proband and relative scores for FSIQ and vocab scores were not modulated by proband age (*p* > 0.5). *FSIQ* Full-Scale Intelligence Quotient, *SRS-2* Social Responsiveness Scale Second Edition, *SRS-2 Awareness* SRS-2 Social Awareness, *SRS-2 Cognition* SRS-2 Social Cognition, *SRS-2 Communication* SRS-2 Social Communication, *SRS-2 Motivation* SRS-2 Social Motivation, *SRS-2 RIRB* SRS-2 Restricted Interests and Repetitive Behaviors, *SRS-2 DSM-5 SCI* SRS-2 DSM-5 Social Communication and Interaction

Regression analyses revealed a statistically significant offset in proband scores relative to family scores for all traits. Observed offsets (i.e., the penetrance of XYY syndrome) varied in magnitude across traits, with the greatest offsets observed for ADHD-related traits (Cohen’s *d* > 2 score elevation), followed by SRS-2 measures of ASD-related traits (median Cohen’s *d* across scales ~ 1.7 score elevation) and FSIQ measures (Cohen’s *d* ~ 1.3 score decrement for FSIQ and vocabulary, and ~ 0.9 matrix reasoning).

### Relationship between proband and family scores for individual traits: preliminary analysis of slopes

Analysis of model slope terms indicated that FSIQ and vocabulary showed by far the closest correspondence between variation in proband and family scores as compared to other scales. The observed slopes for these traits were ~ 0.9 and both statistically indistinguishable from 1, indicating that across the full range of IQ scores observed in our cohort, there was an approximately 1:1 relationship between score differences across probands and score differences across their unaffected relatives (Table 2, Fig. 2a,b). The equivalent slope term for regression analysis of matrix reasoning was statistically indistinguishable from 0 (Table 2, Fig. 2c). Examination of adjusted *R*^2^ estimated from regression analyses indicated that variability in family scores explained approximately 39% of the variance in proband FSIQ, 31% of variance in proband vocabulary scores, and 3% of variance in proband matrix reasoning. Besides FSIQ and vocabulary scores, only one other trait examined had an estimated slope from regression analysis that was statistically significantly different from 0: the SRS-2 social awareness subscale. However, the observed slope for prediction of variation in proband social awareness scores from those in unaffected relatives was negative (− 0.6, Table 2, Fig. 2d), suggesting that greater degrees of impaired social awareness in XYY probands by parent rating tend to be associated with better social awareness in first degree relatives.

### Exploring cross-trait influences of family IQ and perinatal variables

Several measures of proband ASD traits from the SRS-2, including total score, social communication, and DSM-5 social communication and interaction scores, were significantly associated with family IQ in models that also accounted for family scores on the corresponding ASD trait (Additional file 4). For all of these traits, greater family FSIQ scores were associated with reduced SRS-2 scores in probands. These extended models including family IQ explained approximately four times greater variance in the named proband SRS-2 traits than models which only included the corresponding SRS-2 trait in family members' measures. However, the absolute proportion of variance that could be predicted for proband scores remained low (average ~ 5%, Additional file 4). Further extending multiple linear regression models to include family SES and a set of perinatal factors (see “Methods” section) did not yield significantly greater prediction for any proband traits measured in this study (Additional file 4).

### Exploratory cluster analysis of all pairwise trait-relative relationships

**Fig. 3:**
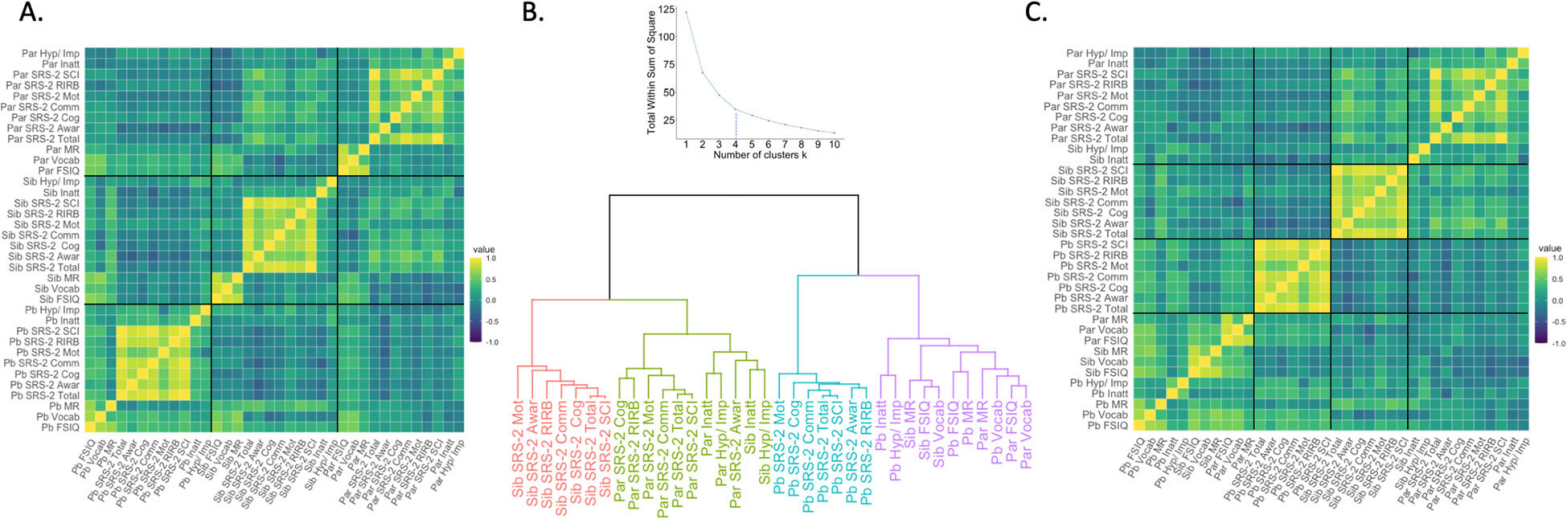
Cluster analyses. All measures are z-scored, and IQ measures are inverted such that a higher IQ score signifies more impairment. **a** Family cognitive and behavioral phenotype correlation matrix organized by family member. **b** Hierarchical clustering tree based on unsupervised clustering algorithm and elbow of within cluster sum of squares T 4. **c** Family cognitive and behavioral phenotype correlation matrix reorganized by unsupervised clustering algorithm. *Proband (Pb). Sibling (Sib). Parent (Par). Full Scale Intelligence Quotient (FSIQ). Social Responsiveness Scale Second Edition (SRS-2). SRS-2 Social Awareness (Social Awar). SRS-2 Social Cognition (Social Cog). SRS-2 Social Communication (Social Comm). SRS-2 Social Motivation (Social Mot). SRS-2 Restricted Interests and Repetitive Behaviors (SRS-2 RIRB). SRS-2 DSM-5 Social Communication and Interaction (SRS-2 SCI). ADHD Inattentive Symptoms (Inatt). ADHD Hyperactive-Impulsive Symptoms (Hyp/Imp)*

The pairwise correlation matrix across all measures for all family members is shown in Fig. 3. Ordering trait measures by their family member of origin created a 3 × 3 quadrant structure, highlighting three distinct classes of intra-familial trait comparisons: parent-proband, parent-proband’s sibling and proband-sibling (Fig. 3a). Qualitatively, this correlation matrix shows that (i) inter-trait correlations tend to be higher within an individual than between individuals, and (ii) trait correlations tend to be lower between parents and XYY probands than between parents and unaffected siblings of XYY probands. Unsupervised clustering of this full pairwise relative-trait correlation matrix according to an empirically selected 4 cluster solution (Fig. 3b) separated out the following subsets of traits across family members: (i) a “Family IQ and Proband ADHD-trait” cluster, which includes all IQ measures (FSIQ, vocabulary, and matrix reasoning) for all family members, proband ADHD inattentive and hyperactive-impulsive symptoms, (ii) a “Parent Psychopathology and Sibling ADHD-trait” cluster of all parent SRS-2 measures, parent ADHD inattentive and hyperactive-impulsive symptoms, and sibling ADHD inattentive and hyperactive-impulsive symptoms, (iii) a “Sibling Psychopathology” cluster including all sibling SRS-2 measures, and (iv) a “Proband Psychopathology” cluster of all proband SRS-2 measures (Fig. 3c). The hierarchical relationship between these four clusters (Fig. 3b) supports the qualitative impression from Fig. 3a by revealing a higher-order set of two clusters: Proband Psychopathology and Family IQ vs. Parent Psychopathology and Sibling Psychopathology.

## Discussion

Our study develops and applies a set of complementary methods for prediction of outcome variability in aneuploidy and CNV carriers based on family data. By using these methods to model data from families with male probands carrying an extra Y chromosome, we (i) refine insights into the impact of XYY on several aspects of early cognitive and behavioral development, and (ii) show the general utility of family-based analyses for resolving interdependencies between neurobehavioral outcomes in aneuploidy or CNV carriers, and neurobehavioral profiles in their first degree, non-carrier relatives. We address the implications of our results below and consider important steps for future work.

The correlation analyses in this study indicate that the degree of coherence between trait variability across aneuploidy-carrying probands and their unaffected family members can vary greatly across traits. The strongest positive proband-family trait correlations were seen for FSIQ (0.63). Similar magnitudes of proband-family FSIQ correlations have also been reported in several other aneuploidies and CNVs [22q11.2.2 deletion syndrome: [10], 16p11.2.2 deletion syndrome: [4], Down syndrome: [35], and Klinefelter syndrome [36]], as well as in the general population [10, 37]. This suggests that although most neuropsychiatric aneuploidies and CNVs significantly impact FSIQ, many do so without disrupting the other sources of variance that underpin the well-documented familiality of these traits in the general population [38]. In other words, the causal pathways that mediate the negative impact of many aneuploidies and CNVs on FSIQ may be partly distinct from those that explain intrafamilial FSIQ correlations. This principle may not hold equally for vocabulary and matrix reasoning, however—although formal tests of this notion must await direct comparisons in larger samples than ours of family-proband correlations for different traits. In our present study of XYY syndrome, and in past studies of XXY syndrome and 16p11.2 deletion disorder, the correlation between probands and first-degree relatives was stronger for verbal than non-verbal subcomponents of general cognitive ability [4, 36].

Further, in contrast to the robustly positive proband-family correlations for FSIQ, we observe near-zero or negative proband-family correlations for several SRS-2 traits in XYY syndrome (0.088 > *r* > − 0.26). This finding is surprising given that previous reports have found strong and positive proband-family correlations for social impairment in 16p11.2.2 deletion syndrome [4], and in the general population (e.g., parent-child *r* ~ 0.35 [37]). One potential explanation for these findings is a differential rater effect across studies or syndromes. For example, variation in parental social functioning may influence how parents rate the social behavior of affected offspring (e.g., parents with greater social awareness are more sensitive to social impairments), and these sorts of effects may vary by the nature of social dysfunction seen in different neurogenetic disorders. Another potential explanation is that the uncoupling of child from parent SRS-2 scores is driven in part by aspects of the underlying biology of XYY syndrome, such that carriage of an extra Y-chromosome introduces independent sources of variance in social functioning that disrupt or overwhelm the influence of familial factors. For example, given that psychopathology in XYY syndrome has been shown to affect the specificity of the SRS-2 screening form [3], the specificity of SRS-2 ratings may differ between genetic disorders with dissimilar profiles of psychopathology—thereby leading to differing proband-parent correlations in SRS-2 scores. The variability of the relationship between family and XYY probands across different traits, and the differences between findings in XYY and gene dosage disorders like 16p11.2 deletion syndrome, suggests that the accuracy of family data in predicting proband outcomes is likely to be both trait- and disorder-specific. Taken together, our findings suggest that knowing family scores for certain traits, such as FSIQ and vocabulary scores, is useful for predicting proband trait variation across aneuploidy and CNV disorders, but that other traits, such as social impairment, can show highly variable proband-family correlations in different aneuploidy and CNV disorders.

Our findings also emphasize the added value of modeling proband-family trait interrelationships within a regression framework [4, 10, 36]. Specifically, by using regression to estimate proband offsets relative to family members versus the general population, we derived more refined estimates of the penetrance of XYY syndrome. This refinement is achieved because, (i) recruitment biases may enrich probands for particular background genetic and environmental factors that influence traits that are also impacted by XYY syndrome, and (ii) family-based offsets estimate the penetrance of XYY syndrome while controlling for the background genetic and environmental factors that probands share with their family members. For some traits, such as IQ and ADHD, our family-based models estimated offsets that differ from prior studies where trait scores in XYY syndrome were compared to those from recruited controls or standardized instrument distributions [33, 39]. We observed the largest offsets for ADHD-related traits, followed by ASD-related traits and FSIQ/vocabulary scores.

In addition to allowing offset estimation, regression approaches also provide a quantitative framework for estimation of proband scores given known family scores. However, the magnitudes of these slopes were not as well estimated as the proband offsets, and given the small sample size of our cohort, results from these regression slope analyses must be considered provisional in nature. By analyzing these slopes, our study reveals that the average magnitude of FSIQ reduction in XYY probands relative to their first-degree relatives is expected to be the same (~ 1.3 Cohen’s *d* effect size) across different levels of proband and family IQ. This finding indicates that any potential ascertainment biases that might enrich for recruitment of families with unusually high or low FSIQs should not bias estimation of the penetrance of XYY for FSIQ reduction (although may still bias estimation for other neurobehavioral measures).

Regression analysis also facilitates multivariate modeling of proband outcomes from family data, which we harnessed to test for potential moderating effects of family FSIQ and perinatal variables on other proband-family trait interrelationships. These analyses revealed that there is a negative association between family IQ and proband SRS-2 scores: a higher family IQ score provides a protective effect for some aspects of social responsiveness. This finding demonstrates the need to examine cross-measure, family-to-proband relationships to better predict proband outcomes in the future. Multiple linear regression models also suggested that variability in XYY proband outcomes is not significantly related to variability in familial SES and perinatal variables. Further expansion of such multiple linear regression methods would help to more systematically determine the limits of our capacity to predict variation in proband outcomes from family phenotypic data.

Finally, our framework applies clustering analysis to resolve the effect of an additional Y-chromosome on coherence within and between family members across several cognitive and behavioral measures. In this context, clustering provides an analytically efficient means of describing the architecture of trait variation within families. Observing cluster composition can indicate the extent to which clustering of traits within a family is governed by carrier status (e.g., clusters separate carriers from others regardless of trait) as compared to the phenotype being considered (e.g., clusters separate IQ from other traits regardless of the family member being measured). In application to XYY probands and their family members, we observe co-clustering of traits into 4 broad groups (i) Family IQ and Proband ADHD-traits, (ii) Parent Psychopathology and Sibling ADHD-traits, (iii) Sibling Psychopathology, and (iv) Proband Psychopathology. All of the SRS-2 measures for a given family member cluster together, which is expected due to the high internal consistency of the SRS-2 [40]. The clustering of ADHD-traits in parents and unaffected siblings was also found in previous reports of children with ADHD [41]. Notably, proband ADHD-traits clustered with family IQ measures, suggesting that the sources of ADHD-trait variation may differ between unaffected siblings and the XYY probands. While overall clustering patterns suggest a greater coherence between parent and sibling psychopathology compared to parent and proband psychopathology, all of the family IQ measures cluster together, suggesting that carriage of an extra Y-chromosome may disrupt coherence between probands and family members for psychopathology, but not for IQ measures. We anticipate that similar applications of clustering to family-based phenotypic data may help to reveal traits in relatives that are most closely coupled to trait variation in carriers, and to specify the effects of applying different dimension reduction techniques to familial phenotypic data.

Our findings should be considered in light of several limitations and caveats. First, because the families in our cohort were not identified through a population-based sampling frame, they may not be fully representative of the full range of outcomes and background factors seen in XYY-probands and their first-degree relatives. Second, our study is cross-sectional in design and therefore cannot resolve potentially age-varying proband-family interrelationships. Third, observed correlations between rating scale scores can be influenced by methodological aspects which our study design does not directly model, such as parent versus child [42], or mother versus father [43] rater effects. Relatedly, our study design is not able to disambiguate the many potential sources of observed correlations between parent and child traits, which could include highly contrasting mechanisms—i.e., shared genetic determinants of IQ variation between probands and parents versus high parental psychopathology being driven by high caregiver strain, which in turn relates to proband psychopathology. In a similar vein, the variable contribution of unaffected relatives to estimated trait scores across different families means that the observed trait correlations between probands and families reflects a composite of different degrees of genetic relatedness. However, our findings from naturalistic estimation of trait correspondence between probands and their family members provide a valuable reference-point when contemplating how such approaches might be used in practice to improve prediction of proband outcomes from family data in practice. Finally, our modest sample size necessarily places limits on our power to confidently detect certain phenomena—such as those modelled by the slope terms in family-proband regression analyses. Future analyses in larger cohorts will help to address this limitation and also to directly test the reproducibility of our findings.

## Conclusions

Given the extensive phenotypic variation observed in individuals with aneuploidies and pathogenic CNVs, there is a need to build models that will allow for a better prediction of outcomes in individual carriers. Our study expands the suite of tools for modeling trait interrelationships between aneuploidy/CNV carriers and their unaffected family members. The utility of these tools is demonstrated in application to XYY syndrome, where we find that (i) as reported for several other gene dosage disorders, FSIQ and vocabulary scores show strong correlation between XYY probands and family members, (ii) the proband-family coupling in ASD-related traits that has been reported in CNV disorders appears to be lost in XYY syndrome, (iii) family FSIQ shows a statistically significant “cross-trait” relationship with the severity of ASD-related symptoms in XYY probands, and (iv) carriage of an extra Y-chromosome may weaken correlations that are otherwise seen between parent and offspring psychopathology, but does not weaken this correlation for IQ. While the initial application of this model was in XYY syndrome, this framework can also be used to predict outcomes in probands with other chromosomal aneuploidies and copy number variants.

## Supplementary Information

**Additional file 1.** Full clinical and demographic information.

**Additional file 2.** Testing and Questionnaire Distribution.

**Additional file 3.** Proband to Family Full Univariate Analysis. (PPTX 371 kb)

**Additional file 4.** Multiple linear regression models to predict cognitive and behavioral proband outcomes.

## Data Availability

The XYY data are registered with ClinicalTrials.gov under the name “89-M-0006: Brain Imaging of Childhood Onset Psychiatric Disorders, Endocrine Disorders and Healthy Controls.”

## Abbreviations

ADHD: Attention deficit hyperactivity disorder
ASD: Autism spectrum disorder
CAARS-S:L: Conners’ Adult ADHD Rating Scales-Self-Report: Long Version
CNVs: Copy number variations
FSIQ: Full-Scale Intelligence Quotient
IQ: Intelligence quotient
SRS-2: Social Responsiveness Scale Second Edition
SES: Socioeconomic status

## References

[1] Kirov G (2015). CNVs in neuropsychiatric disorders. Hum Mol Genet..

[2] Malhotra D, Sebat J (2012). CNVs: harbingers of a rare variant revolution in psychiatric genetics. Cell..

[3] Joseph L, Farmer C, Chlebowski C, Henry L, Fish A, Mankiw C (2018). Characterization of autism spectrum disorder and neurodevelopmental profiles in youth with XYY syndrome. J Neurodev Disord..

[4] Moreno-De-Luca A, Evans DW, Boomer KB, Hanson E, Bernier R, Goin-Kochel RP (2015). The role of parental cognitive, behavioral, and motor profiles in clinical variability in individuals with chromosome 16p11.2 deletions. JAMA Psychiatry.

[5] McDonald-McGinn DM, Sullivan KE (2011). Chromosome 22q11.2 deletion syndrome (DiGeorge syndrome/velocardiofacial syndrome). Medicine.

[6] Olsen L, Sparsø T, Weinsheimer SM, Dos Santos MBQ, Mazin W, Rosengren A (2018). Prevalence of rearrangements in the 22q11.2 region and population-based risk of neuropsychiatric and developmental disorders in a Danish population: a case-cohort study. Lancet Psychiatry..

[7] Zufferey F, Sherr EH, Beckmann ND, Hanson E, Maillard AM, Hippolyte L (2012). A 600 kb deletion syndrome at 16p11.2 leads to energy imbalance and neuropsychiatric disorders. J Med Genet..

[8] Constantino JN, Davis SA, Todd RD, Schindler MK, Gross MM, Brophy SL (2003). Validation of a brief quantitative measure of autistic traits: comparison of the social responsiveness scale with the autism diagnostic interview-revised. J Autism Dev Disord..

[9] Ottesen AM, Aksglaede L, Garn I, Tartaglia N, Tassone F, Gravholt CH (2010). Increased number of sex chromosomes affects height in a nonlinear fashion: a study of 305 patients with sex chromosome aneuploidy. Am J Med Genet A..

[10] Olszewski AK, Radoeva PD, Fremont W, Kates WR, Antshel KM (2014). Is child intelligence associated with parent and sibling intelligence in individuals with developmental disorders? An investigation in youth with 22q11.2 deletion (velo-cardio-facial) syndrome. Res Dev Disabil..

[11] Goodman R (1995). The relationship between normal variation in IQ and common childhood psychopathology: a clinical study. Eur Child Adolesc Psychiatry..

[12] Keyes KM, Platt J, Kaufman AS, McLaughlin KA (2017). Association of fluid intelligence and psychiatric disorders in a population-representative sample of US adolescents. JAMA Psychiatry..

[13] tyler. AXYS | The association for X and Y chromosome variations. The Association for X and Y Chromosome Variations. [cited 2020 Apr 3]. Available from: http://genetic.org

[14] Chen AT, Chan YK, Falek A (1971). The effects of chromosome abnormalities on birth weight in man. I. Sex chromosome disorders. Hum Hered..

[15] Constantino JN, Gruber CP. Social Responsiveness Scale Second Edition (SRS-2): Manual. Western Psychological Services (WPS); 2012.

[16] Conners CK (2008). Conners 3rd edition: Manual.

[17] Conners CK, Erhardt D, Sparrow E. Conners’ Adult ADHD Rating Scales--Self-Report: Long Version (CAARS--S: L). Toronto, Ontario, Canada: Multi-Health Systems. documents.acer.org; 2002; Available from: https://documents.acer.org/caars-self-l-int1.pdf

[18] Colvin M, McGuire W, Fowlie PW (2004). Neurodevelopmental outcomes after preterm birth. BMJ..

[19] Merikangas AK, Calkins ME, Bilker WB, Moore TM, Gur RC, Gur RE (2017). Parental age and offspring psychopathology in the Philadelphia Neurodevelopmental Cohort. J Am Acad Child Adolesc Psychiatry..

[20] Oommen SP, Santhanam S, John H, Roshan R, Padankatti C, Swathi TO (2019). Neurodevelopmental Outcomes of Very Low Birth Weight Infants at 18-24 Months, Corrected Gestational Age in a Tertiary Health Centre: A Prospective Cohort Study. J Trop Pediatr..

[21] Hackman DA, Farah MJ, Meaney MJ (2010). Socioeconomic status and the brain: mechanistic insights from human and animal research. Nat Rev Neurosci..

[22] Thorndike RL (1953). Who belongs in the family?. Psychometrika..

[23] RStudio | Open source & professional software for data science teams. [cited 2020 Apr 3]. Available from: http://www.rstudio.com/.

[24] Henry L, Wickham H. purrr: functional programming tools. R package version 0.2. 4. 2017. Available from: https://CRAN.R-project.org/package=purrr

[25] Wickham H, Francois R, Henry L, Müller K. dplyr: a grammar of data manipulation. R package version 0.4. 3. R Found Stat Comput, Vienna https://CRAN R-project org/package= dplyr. 2015; Available from: https://CRAN.R-project.org/package=dplyr

[26] CRAN - Package magrittr. [cited 2020 Apr 3]. Available from: https://CRAN.R-project.org/package=magrittr

[27] Wickham H. Getting Started with ggplot2. Use R! 2016. p. 11–31. Available from: 10.1007/978-3-319-24277-4_2

[28] Garnier S. viridis: default color maps from “matplotlib”. 2016. R package version 0.3. 4. 2017. Available from: https://CRAN.R-project.org/package=viridis

[29] Galili T, O’Callaghan A, Sidi J, Sievert C (2018). heatmaply: an R package for creating interactive cluster heatmaps for online publishing. Bioinformatics..

[30] Maechler M, Rousseeuw P, Struyf A, Hubert M, Hornik K (2012). Cluster: cluster analysis basics and extensions. R Package Version.

[31] Kassambara A, Mundt F. F. factoextra: extract and visualize the results of multivariate data analyses. R package version 1.0. 3. Recuperado de https://CRAN. R-project. org/package= factoextra. Acesso em; 2017. Available from: https://CRAN.R-project.org/package=factoextra

[32] Printzlau F, Wolstencroft J, Skuse DH (2017). Cognitive, behavioral, and neural consequences of sex chromosome aneuploidy. J Neurosci Res..

[33] Ross JL, Roeltgen DP, Kushner H, Zinn AR, Reiss A, Bardsley MZ (2012). Behavioral and social phenotypes in boys with 47,XYY syndrome or 47,XXY Klinefelter syndrome. Pediatrics. Am Acad Pediatrics.

[34] Ross JL, Tartaglia N, Merry DE, Dalva M, Zinn AR (2015). Behavioral phenotypes in males with XYY and possible role of increased NLGN4Y expression in autism features. Genes Brain Behav..

[35] Fraser FC, Sadovnick AD. Correlation of IQ in subjects with Down syndrome and their parents and sibs. J Ment Defic Res. 1976;20(3):179–82. 10.1111/j.1365-2788.1976.tb00942.x. 10.1111/j.1365-2788.1976.tb00942.x 135090

[36] Netley C (1987). Predicting intellectual functioning in 47,XXY boys from characteristics of sibs. Clin Genet..

[37] Bouchard TJ, McGue M (1981). Familial studies of intelligence: a review. Science..

[38] Devlin B, Daniels M, Roeder K (1997). The heritability of IQ. Nature..

[39] Leggett V, Jacobs P, Nation K, Scerif G, Bishop DVM (2010). Neurocognitive outcomes of individuals with a sex chromosome trisomy: XXX, XYY, or XXY: a systematic review. Dev Med Child Neurol..

[40] Bölte S, Poustka F, Constantino JN (2008). Assessing autistic traits: cross-cultural validation of the social responsiveness scale (SRS). Autism Res..

[41] Margari F, Craig F, Petruzzelli MG, Lamanna A, Matera E, Margari L (2013). Parents psychopathology of children with Attention Deficit Hyperactivity Disorder. Res Dev Disabil..

[42] Hope TL, Adams C, Reynolds L, Powers D, Perez RA, Kelley ML (1999). Parent vs. Self-Report: Contributions Toward Diagnosis of Adolescent Psychopathology. J Psychopathol Behav Assess..

[43] Jensen PS, Traylor J, Xenakis SN, Davis H (1988). Child psychopathology rating scales and interrater agreement: I. Parents’ gender and psychiatric symptoms. J Am Acad Child Adolesc Psychiatry..

