# Supplementary material for: Modeling familial predictors of proband outcomes in neurogenetic disorders: initial application in XYY syndrome
Source: J Neurodev Disord. 2021 Mar 22;13:12. doi: 10.1186/s11689-021-09360-7 (PMC7986517; doi:10.1186/s11689-021-09360-7)
Supplement: Supplementary file 1 — Additional file 1. Full clinical and demographic information. [file 11689_2021_9360_MOESM1_ESM.docx]

**Additional File 1. Full clinical and demographic information.**

|  | Probands  (N = 58) | Siblings  (N = 24) | Mothers  (N = 57) | Fathers  (N = 34) |
| --- | --- | --- | --- | --- |
| **Race**  Asian  White  > 1 race | N = 1  N = 56  N = 1 | N = 0  N = 23  N = 1 | N = 0  N = 56  N = 1 | N = 0  N = 33  N = 1 |
| **Ethnicity**  Hispanic  Non-Hispanic | N = 3  N = 55 | N = 2  N = 22 | N = 3  N = 54 | N = 1  N = 33 |
| **Headedness**  Right  Left  Mixed | N = 51  N = 4  N = 3 | N = 19  N = 1  N = 4 | N = 50  N = 0  N = 7 | N = 30  N = 2  N = 2 |
| **Height (cm)** | 158 + 28  (109 – 201) | 152 + 23  (113 - 183) | 165 + 5  (155 - 180) | 179 + 6  (168 - 190) |
| **Weight (kg)** | 60 + 34  (18 – 132) | 54 + 29  (24 - 121) | 85 + 23  (48 - 142) | 100 + 19  (66 - 143) |
| **Tanner Stage**  1  2  3  4  5 | N = 23  N = 7  N = 3  N = 15  N = 10 | N = 9  N = 2  N = 5  N = 3  N = 3 |  |  |
| **Birth Weight (oz)** | 115 + 22  (58 - 160) | 123 + 18  (93 - 155) |  |  |
| **Gestation**  **Period (weeks)** | 38 + 2  (31 - 42) | 39 + 2  (36 - 42) |  |  |
| **47,XYY Diagnosis**  Prenatal/ Birth  Postnatal | N = 22  N = 36 |  |  |  |
| **Age at XYY Dx (years)** | 3.4 + 4.7  (-0.5 - 16) |  |  |  |
| **Specialized Services**  504 Plan  Aide  IEP  None  Other | N = 3  N = 2  N = 38  N = 1  N = 14 |  |  |  |

Mean + Standard Deviation (Range) or N = number of participants.
