# Supplementary material for: Modeling familial predictors of proband outcomes in neurogenetic disorders: initial application in XYY syndrome
Source: J Neurodev Disord. 2021 Mar 22;13:12. doi: 10.1186/s11689-021-09360-7 (PMC7986517; doi:10.1186/s11689-021-09360-7)
Supplement: Supplementary file 2 — Additional file 2. Testing and Questionnaire Distribution. [file 11689_2021_9360_MOESM2_ESM.docx]

**Additional File 2. Testing and Questionnaire Distribution.**

| Test/ Questionnaire | Probands | Siblings | Mothers | Fathers |
| --- | --- | --- | --- | --- |
| **Wechsler Intelligence Scales**  WISC-V  WPPSI-IV  WAIS-IV  WASI-II | N = 32  N = 13  N = 10  N = 3 | N = 1  N = 23 | N = 57 | N = 34 |
| **Social Responsiveness Scale Second Edition (SRS-2)**  SRS-2 School-age report  *(for 4-18-year-olds, completed by parents)*  SRS-2 Adult report  *(for 19+ year-olds,*  *completed by parents)*  SRS-2 Self-report | N = 48  N = 10 | N = 19  N = 5 | N = 57 | N = 34 |
| **ADHD-trait Measures**  Conners 3 - Parent Edition  *(for 6-18-year-olds, completed by parents)*  CAARS-S:L  *(for 18+ year-olds, self-report)* | N = 46  N = 10 | N = 18  N = 5 | N = 57 | N = 34 |

*Wechsler Intelligence Scale for Children, 5th Edition (WISC-V), Wechsler Preschool and Primary Scale of Intelligence, 4th Edition (WPPSI-IV), Wechsler Adult Intelligence Scale, 4th edition (WAIS-IV), Wechsler Abbreviated Scale of Intelligence, 2nd Edition (WASI-II). Conners’ Adult ADHD Rating Scales- Self-Report: Long Version (CAARS-S:L).*
