# Supplementary figures and images for: Modeling familial predictors of proband outcomes in neurogenetic disorders: initial application in XYY syndrome
Source: J Neurodev Disord. 2021 Mar 22;13:12. doi: 10.1186/s11689-021-09360-7 (PMC7986517; doi:10.1186/s11689-021-09360-7)

## Slide 1
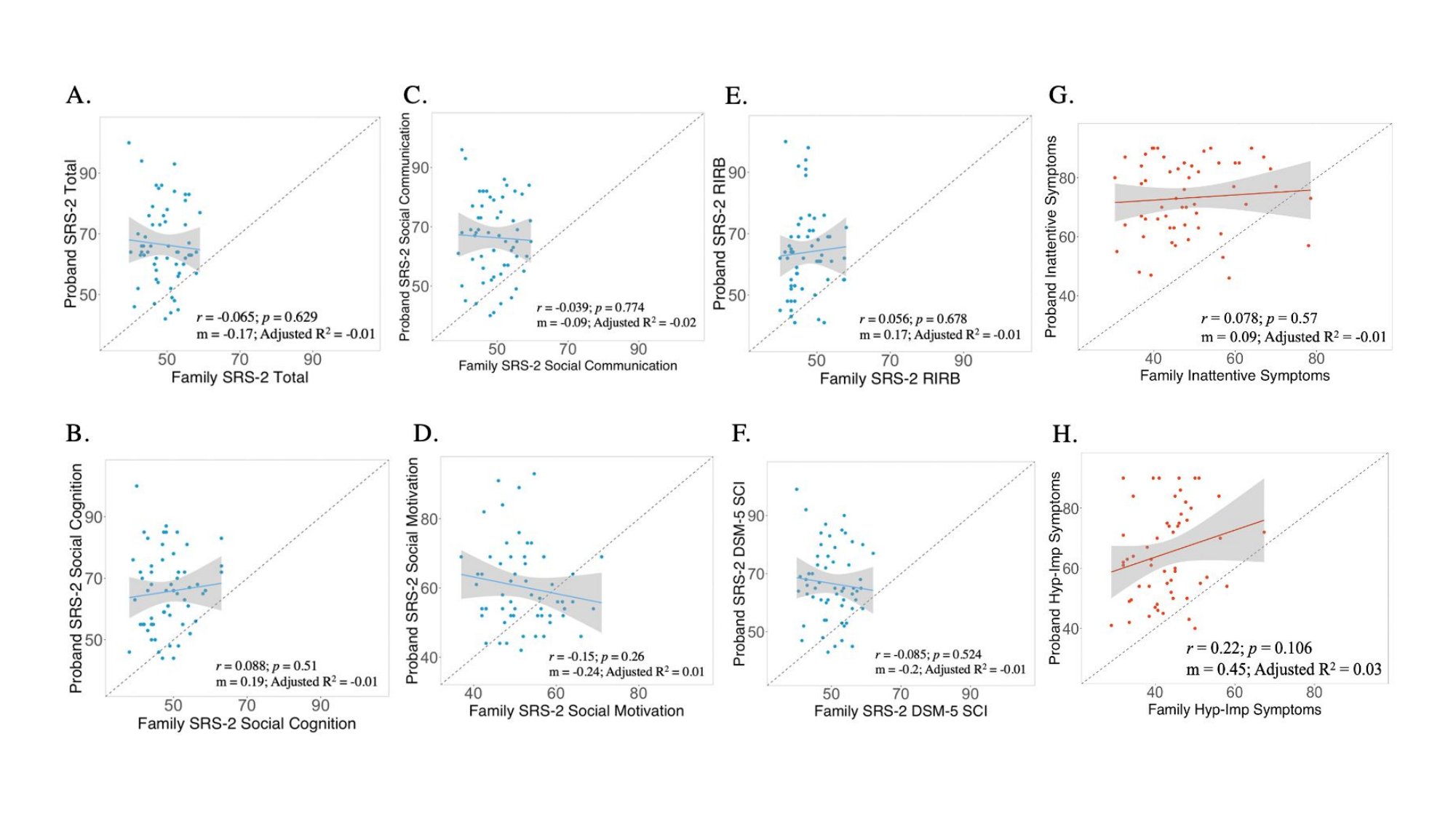

Supplement: Supplementary file 3 — Additional file 3. Proband to Family Full Univariate Analysis. (PPTX 371 kb) [file 11689_2021_9360_MOESM3_ESM.pptx]
