# Supplementary material for: Modeling familial predictors of proband outcomes in neurogenetic disorders: initial application in XYY syndrome
Source: J Neurodev Disord. 2021 Mar 22;13:12. doi: 10.1186/s11689-021-09360-7 (PMC7986517; doi:10.1186/s11689-021-09360-7)
Supplement: Supplementary file 4 — Additional file 4. Multiple linear regression models to predict cognitive and behavioral proband outcomes. [file 11689_2021_9360_MOESM4_ESM.docx]

**Additional File 4. Multiple linear regression models to predict cognitive and behavioral proband outcomes.**

| Proband Feature | Equivalent Family Measure  in Model 1 | Family FSIQ  in Model 1 | Adjusted R^2^ for Univariate Model | Adjusted R^2^ for Model 1 | Adjusted R^2^ for Model 2 | Adjusted R^2^: Model 1 to Model 2 |
| --- | --- | --- | --- | --- | --- | --- |
| FSIQ | NA | *t* = 6.051  ***p* = 1.5E-07*** | R^2^ = 0.40 | R^2^ = 0.40 | R^2^ = 0.41 | *p* = 0.30 |
| Vocabulary | *t* = 1.428  *p* = 0.161 | *t* = 1.120  *p* = 0.269 | R^2^ = 0.31 | R^2^ = 0.32 | R^2^ = 0.25 | *p* = 0.91 |
| Matrix Reasoning | *t* = -0.561  *p* = 0.577 | *t* = 1.758  *p* = 0.086 | R^2^ = 0.04 | R^2^ = 0.07 | R^2^ = 0.16 | *p* = 0.08 |
| SRS-2 Total | *t* = -0.393  *p* = 0.696 | *t* = -2.028  ***p* = 0.048** | R^2^ = -0.02 | R^2^ = 0.04 | R^2^ = 0.03 | *p* = 0.51 |
| SRS-2 Aware | *t* = -1.828  *p* = 0.073 | *t* = -1.254  *p* = 0.215 | R^2^ = 0.05 | R^2^ = 0.06 | R^2^ = 0.06 | *p* = 0.42 |
| SRS-2 Cognition | *t* = 0.714  *p* = 0.478 | *t* = -1.634  *p* = 0.108 | R^2^  < -0.01 | R^2^ = 0.03 | R^2^ = -0.02 | *p* = 0.78 |
| SRS-2 Comm | *t* = -0.148  *p* = 0.883 | *t* = -2.356  ***p* = 0.022** | R^2^ = -0.02 | R^2^ = 0.06 | R^2^ = 0.03 | *p* = 0.64 |
| SRS-2 Mot | *t* = -1.099  *p* = 0.277 | *t* = -1.794  *p* = 0.08 | R^2^ < 0.01 | R^2^ = 0.05 | R^2^ = 0.09 | *p* = 0.22 |
| SRS-2 RIRB | *t* = 0.261  *p* = 0.795 | *t* = -1.250  *p* = 0.217 | R^2^ = -0.02 | R^2^ < -0.01 | R^2^ = -0.03 | *p* = 0.58 |
| SRS-2 SCI | *t* = -0.557  *p* = 0.58 | *t* = -2.229  ***p* = 0.030** | R^2^ = -0.01 | R^2^ = 0.06 | R^2^ = 0.04 | *p* = 0.53 |
| ADHD Inatt | *t* = 0.517  *p* = 0.608 | *t* = -0.718  *p* = 0.476 | R^2^ = -0.02 | R^2^ = -0.02 | R^2^ = -0.04 | *p* = 0.53 |
| ADHD Hyp-Imp | *t* = 1.343  *p* = 0.185 | t = -2.003  *p* = 0.051 | R^2^ = 0.02 | R^2^ = 0.08 | R^2^ = 0.01 | *p* = 0.88 |

The *t* and *p*-values are reported for both independent variables in model 1. Adjusted R squared values for the univariate analysis, model 1, and model 2 are reported. The predictors in model 1 were family FSIQ and the family measure equivalent to the predicted proband measure. The predictors in model 2 were the same as model 1, as well as SES, MacArthur US Ladder, birth weight, gestation period, and maternal age. The *p*-values under family FSIQ are equivalent to the ANOVA *p*-values between the univariate model and model 1. The *p*-values for the ANOVA test between multiple linear regression models 1 and 2 are in the final column. (*) indicates survived multiple hypothesis testing Bonferroni correction. *Full Scale Intelligence Quotient (FSIQ). Social Responsiveness Scale Second Edition (SRS-2). SRS-2 Social Awareness (SRS-2 Aware). SRS-2 Social Cognition (SRS-2 Cognition). SRS-2 Social Communication (SRS-2 Comm). SRS-2 Social Motivation (SRS-2 Mot). SRS-2 Restricted Interests and Repetitive Behaviors (SRS-2 RIRB). SRS-2 DSM-5 Social Communication and Interaction (SRS-2 SCI). ADHD Inattentive (ADHD Inatt). ADHD Hyperactive-Impulsive (ADHD Hyp-Imp).*
